# Total endoscopic sublay mesh repair for umbilical hernias

**DOI:** 10.1097/MD.0000000000026334

**Published:** 2021-06-25

**Authors:** Xiao-yan Cai, Ke Chen, Yu Pan, Xiao-yan Yang, Di-yu Huang, Xian-fa Wang, Qi-long Chen

**Affiliations:** Department of General and Minimally Invasive Surgery, Sir Run Run Shaw Hospital, College of Medicine, Zhejiang University, Hangzhou, Zhejiang, China.

**Keywords:** endoscopy, mesh, minimally invasive surgery, sublay, umbilical hernia

## Abstract

Umbilical hernias constitute some of the most common surgical diseases addressed by surgeons. Endoscopic techniques have become standard of care together with the conventional open techniques for the treatment of umbilical hernias. Several different approaches were described to achieve laparoscopic sublay repair.

We prospectively collected and reviewed the medical records of 10 patients with umbilical hernias underwent total endoscopic sublay repair (TES) at our institution from November 2017 to November 2019. All operations were performed by a same surgical team. The demographics, intraoperative details, and postoperative complications were evaluated.

All TES procedures were successfully performed without conversion to an open operation. No intraoperative morbidity was encountered. The average operative time was 109.5 minutes (range, 80–140 minutes). All the patients resumed an oral diet within 6 hours after the intervention. The mean time to ambulation was 7.5 hours (range, 4–14 hours), and mean postoperative hospital stay was 2.2 day (range, 1–4 days). One patient developed postoperative seroma. No wound complications, chronic pain, or recurrence were registered during the follow-up.

Initial experiences with this technique show that the TES is a safe, and effective procedure for the treatment of umbilical hernias.

## 1 Introduction

Ventral and incisional hernia repair is one of the common operations addressed in everyday clinical practice.^[1]^ Umbilical and paraumbilical hernias account for 10% to 12% of abdominal wall hernias.^[2]^ Surgical repair is the only method to treatment. According to the literature, incorporation of mesh for hernia repair has reduced recurrence rates from 63% to 16%.^[3,4]^ With mesh being increasingly used, there is debate as to which plane of mesh placement has the best outcomes.^[5,6]^ The Rives–Stoppa procedure, also known as retro-muscular or sublay repairs, in which the retro-rectus space must be meticulously developed, has some of the most technically challenges due to the necessity of creating the plane for placement of the mesh.^[7]^ However, despite the added difficulties, there is increasing evidence to show that sublay mesh location had lower complication rates than other mesh locations.^[5,6,8,9]^ The retrorectus space serves as a well-vascularized position where mesh prostheses become incorporated and sublay repair has benefits at both molecular and pure mechanical levels.^[10]^ The retro-rectus and preperitoneal mesh placement are recognized as the safest options for hernia repair.^[11]^ Importantly, a thorough understanding of the sublay repair can benefit surgeons performing the operation as well as patients suffering from the morbidity of ventral and incisional hernias.^[12]^

Minimally invasive surgery (MIS) has been the main direction established in terms of surgical development in the 21st century.^[13]^ MIS for repair of ventral/incisional hernias have dramatically changed the care of hernia disease since laparoscopy was introduced in the early 1990s.^[14]^ Since then, studies have concluded that laparoscopic approach to ventral hernia repair (LVHR) produce earlier discharge from the hospital, potentially faster recovery and equivalent recurrence rates in comparison to open approaches.^[15–17]^ The fundamental theoretic assumption of laparoscopic ventral hernia repair is intraperitoneal placement of mesh with fixation. The potential risks associated with an intraperitoneal mesh and traumatic fixation has not yet been eliminated, such as intestinal adhesion, mesh erosion, and chronic pain. Thus, surgeons tried several different approaches to achieve laparoscopic sublay repair.^[18–20]^ Total endoscopic sublay (TES) mesh repair is similar to the classic total extraperitoneal (TEP) hernioplasty for inguinal hernia, avoiding many of the potential complications associated with peritoneal violation.^[21]^ This study aims to determine whether TES technique is a safe and feasible approach for the repair of umbilical hernia by evaluating the surgical outcomes and summarizing experiences.

## 2 Materials and methods

### 2.1 Patients

Between November 2017 and 2019, a prospective study was designed to assess the feasibility and safety of TES for patients with umbilical hernias admitted to the Department of General and Minimally Invasive Surgery, Sir Run Run Shaw Hospital, China. All the patients were fully informed about the details of the surgical procedure, and written informed consents were taken. The study was approved by the Ethics Committee of Zhejiang University. All procedures were performed by the same surgical team. Preoperative computer tomography (CT) was used in assessment of the hernia defect and abdominal domain. The anatomy of the hernia defect, history of wound infection, and complications are all parts of the initial assessment. The patients’ demographics, intraoperative details (conversion, operative time, and estimated blood loss), and short-term outcomes (morbidity, mortality, and postoperative hospital stay) were evaluated. The VAS was checked at postoperative 24 hours.

### 2.2 Surgical procedure

**Figure 1:**
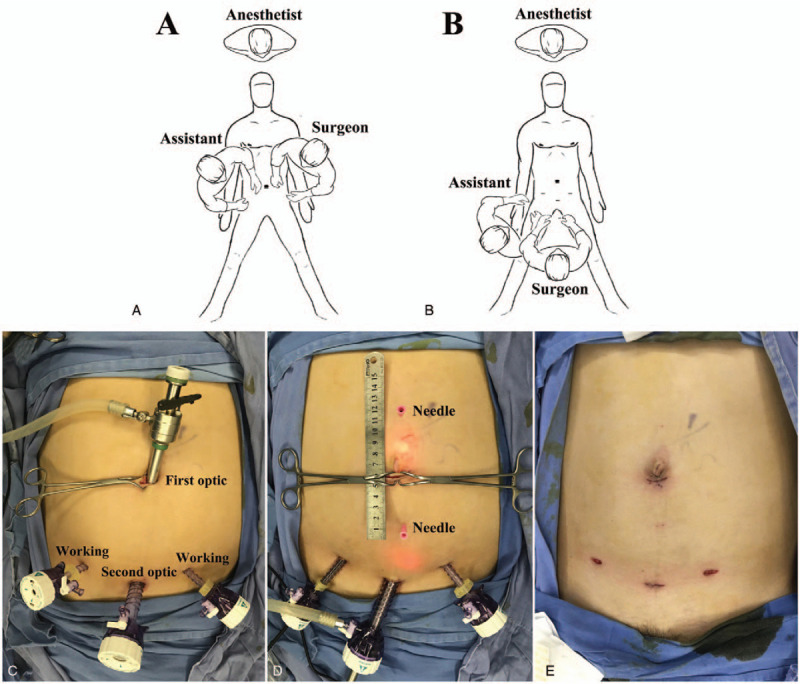
Proper position and body surface. (A) Surgeon position for the isolation of pre-peritoneal space. (B) Surgeon position for the isolation of retro-rectus space. (C) Location of trocars placement and incision. (D) Surgical needles on body surface. (E) Postoperative view of wounds.

**Figure 2:**
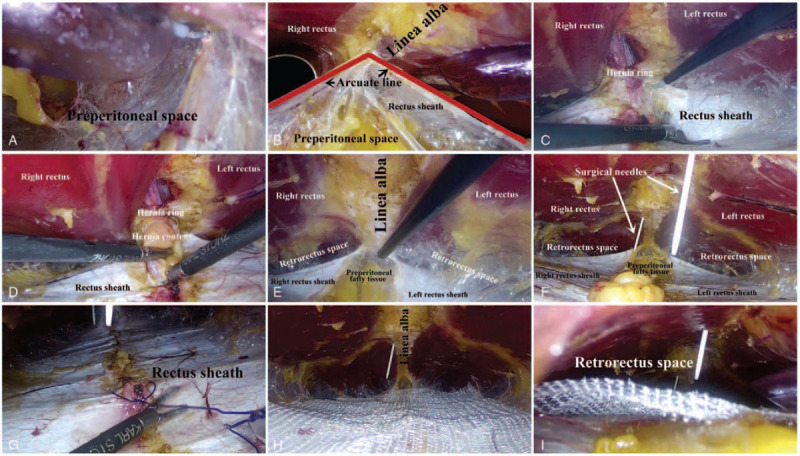
Intraoperative laparoscopic photographs. (A) Isolation of retro-rectus space for trocar insertion. (B) View of the arcuate line. (C) Dissection of retro-rectus space. (E) Hernia sac reduction. (F) View of the surgical needles. (G) Closure of the posterior rectus sheath with suture. (H) Sublay mesh positioning. (I) Pneumoperitoneum release.

A video can be found in supplementary material. The patient was positioned supine with both arms tucked to their sides under general anesthesia. The operating table was placed in Trendelenburg position with legs extended down slightly. An infra-umbilical minimal incision was made (Fig. 1A). Then, the anterior rectus sheath was incised and the rectus muscle was retracted laterally to visualize the posterior rectus sheath (PRS). The first optic trocar was inserted into the retro-rectus space, followed by carbon dioxide insufflation. Direct telescopic dissection was used towards the pubis to create preperitoneal space. The second optic trocar was inserted into the preperitoneal space about 3 cm above the pubic symphysis. After blunt dissection bilaterally (Fig. 2A), 2 5 mm working ports were placed on the bilateral inguinal area under direct vision. Then the surgeon move to stand between the legs of the patient and dissect heading in cephalad direction (Fig. 1B and C). Sharp dissection is then used to cut into the medial aspect of the bilateral retro-rectus space, followed by a combination of sharp and blunt dissection to further develop a uniform retro-rectus space. Care is taken to identify the deep inferior epigastric vessels that runs on the posterior caudal portion of rectus abdominis muscle to prevent injury to it. The arcuate line was an important marker to enter the retro-rectus spaces (Fig. 2B). The PRS were incised close to the middle line with preservation of linea alba on the view roof. Then, the retro-rectus space were dissected toward the level of umbilicus and connected through the midline preperitoneal plane (Fig. 2C). Once the retrorectus space was completely developed, hernia contents were reduced with gentle traction (Fig. 2D). Hernia contents should be fully reduced and keep the umbilical skin intact. The PRS was released approximately 5 cm lateral to the edge of the defect and linea alba (Fig. 2E). Surgical needles were inserted transcutaneously to exactly mark the edges of the fascial defects (Figs. 1D and 2F). Preservation of the linea alba was very important in these processes. Once the incarcerated viscera were reduced the hernia defect was measured at its widest portion. The PRS and hernia ring were closed with 3-0 monofilament barbed sutures by continuous fashion (Fig. 2G). Finally, the tailored macroporous lightweight polypropylene mesh (MPPAM, Ethicon) or Parietex Progrip mesh (Medtronics) was placed assuring complete coverage of the space with an overlap of at least 5 cm in each direction (Fig. 2H). Fibrin glue or sutures were used to fix the mesh in case of defect >3 cm. Pneumoperitoneum was released under direct vision to avoid mesh curling (Figs. 1E and 2I). We recommend abdominal compression binder for 1 month postoperatively.

## 3 Results

Patients’ demographics and hernia characteristics are summarized in Table 1. Ten patients, including 7 men and 3 women, were enrolled in this study. The mean age of the patients was 45.7 years (range, 28–71 years) and the mean body mass index (BMI) was 25.5 kg/m^2^ (range, 20.7–30.3 kg/m^2^). The median American Society of Anesthesiologist (ASA) score was 1. Two cases were incisional hernia in the paraumbilical site. One case was recurrent hernia after suture repair. Intraoperative outcomes and postoperative recoveries are presented at Table 2. The procedures were completed successfully for all the patients without the need for conversion to open surgery. The size of the mesh was correlated with the defect size, with defect width ranging from 2 to 5 cm, defect area ranging from 4 to 25 cm^2^, mesh area ranging from 150 to 300 cm^2^. Only fibrin glue fixation was used in 4 cases to secure the mesh to the posterior sheath. Transfascial suture combined with glue was used in 1 incisional hernia with defect of 5 cm. The average operative time was 109.5 minutes (range, 80–140 minutes), with a mean blood loss of 2.3 mL (range, 2–5 mL). All the patients resumed an oral diet within 6 hours after the intervention. The mean time to ambulation was 7.5 hours (range, 4–14 hours). The mean postoperative hospital stay was 2.2 day (range, 1–4 days). The evaluation of acute pain at 24 hours after surgery by VAS score showed good results: the median value was 2 (range, 1–4). Only one symptomatic seroma was registered (10%) and treated conservatively. No wound complications, chronic pain, and no recurrence were registered during a median follow-up period was of 13 (6–31) months.

**Table 1:** Patients’ demographics and hernia characteristics.

Variable	Value
Number of patients	10
Age, y	45.7 ± 13.1
Gender (male/female)	7:3
BMI index, kg/m^2^	25.5 ± 4.0
ASA score (1:2:3)	6:4:0
Presence of comorbidity (yes: no)	4:6
Hypertension	2
Diabetes mellitus	2
Type of hernia	
Primary hernia	8
Incisional hernia	2

BMI = body mass index.

**Table 2:** Intraoperative outcomes and postoperative recoveries.

Variable	Value
Intraoperative complications	0
Defect width, cm	3.1 ± 1.2
Defect area, cm^2^	10.0 ± 8.4
Mesh area, cm^2^	171.1 ± 33.8
Operative time, min	109.5 ± 13.9
Blood loss, mL	2.3 ± 1.5
Mesh fixation	
None	5 (50%)
Fibrin glue alone	4 (40%)
Fibrin glue and suture	1 (5.3%)
Oral diet, h	6
Ambulation, h	7.5 ± 2.7
Postoperative hospital stay, d	2.2 ± 0.9
Morbidity (seroma)	1 (10%)

## 4 Discussion

The abdominal wall consists of a complex fusion of overlapping layers of muscle and connective tissue designed to contain and protect the abdominal viscera while facilitating rotation and approximation of the thorax with respect to the pelvis. Laparoscopic intraperitoneal onlay mesh (IPOM) repair and open sublay mesh repair are currently the more frequently techniques for the treatment of abdominal wall hernias worldwide.^[9,16,22,23]^ IPOM is an alternative technique that has the advantage of laparoscopic preparation and simple mesh implantation, but it is associated with relevant intra-abdominal early and late complications.^[16,24]^ Despite progress in mesh technology and development of coated meshes designed to lower risk of adhesion formation, the potential risks associated with an intraperitoneal foreign body has not yet been eliminated and traumatic mesh fixation increases the risk of adhesions, visceral damage, nerve injury, and acute and chronic pain.^[22,23]^ One of the alternatives is trans-abdominal preperitoneal approach that has long been used for inguinal hernia repair, now applied also to ventral hernias.^[18]^ However, the primary issues of intraperitoneal access and manipulation are not excluded with this technique and the risk of visceral injury may up to 20%.^[25,26]^ Open repairs are burdened with a major skin incision with extensive tissue dissection that may cause a series of problems such as massive trauma, severe postoperative pain, and surgical site infection, which is closely associated with the requirement to reoperate.^[16,27]^ It is generally accepted that the retromuscular/preperitoneal plane is the ideal location for mesh placement.^[11,12,16,22,23]^ Laparoscopic sublay mesh repair, which grants an ideal, retromuscular, and extraperitoneal mesh position, is thus the logical consequence of efforts in recent years: extraperitoneal placement of foreign material where possible while simultaneously minimizing access-related trauma.^[19]^ The excellent results of laparoscopic hernioplasty for inguinal hernia have confirmed with the highest level of evidence the success of minimally invasive preperitoneal mesh repair.^[28]^ So we tried the TES technique in umbilical and paraumbilical hernia to investigate the feasibility and effectiveness.

According to our preliminary experience, we conclude that the advantages of the TES procedure are as follows: the entire operation is performed under totally laparoscopic manipulation; there is no need for specific instruments, and the technique is highly reproducible and less traumatic for both primary umbilical hernia and paraumbilical hernia. The mesh is placed in the sublay position, keeping the foreign materials out of the abdominal cavity, thus the risk of visceral organ injuries and intraperitoneal foreign-body-related complications were minimized. Reduced postoperative pain is achievable due to mesh placement in the retromuscular space by minimizing the aggressive use of penetrating transfascial fixation, the relationship of which to chronic pain is well established.^[29–33]^ In the retromuscular position, great support strength could be provided by the rectus muscles, which are ventral to the mesh, thus allowing rapid tissue integration without the need for traumatic mesh fixation. Chronic pain and movement limitations are among the main complaints of hernia patients, promoting a cycle of inactivity, weight gain, and progressive loss of function. Therefore, improved life quality is predictable due to less pain.^[34]^ TES repair has lower rates of recurrence and wound complications similar with IPOM, such as surgical site infections, cellulitis, wound dehiscence, delayed healing, seromas, and hematomas.^[5,6,8,35–37]^ Sublay repair is suitable to the obese. Studies showed that patients with a BMI >30 kg/m^2^, sublay repairs are not associated with higher recurrence rates compared with those with a BMI <30 kg/m^2^.^[38,39]^ As there is no need for expensive barrier mesh or fixation tacker, the medical cost is reduced dramatically.

In our center, TES primarily indicated for symptomatic primary umbilical hernia or paraumbilical incisional hernias. Despite limited experience, we consider that TES can be also applied for all kinds of small and medium-sized midline and lateral ventral hernias,^[40,41]^ particularly hypogastric hernia neared to inguinal area. TES is not suitable to manage larger defects due to the constraints of the workspace and the stiff, rigid instruments. Further studies are necessary to determine the optimal indications of these new procedures according to the anatomical situation and hernia pathology.

We have summed up the following experience in practice: as to patients position, we suggested supine position with legs apart, then the table should be given a 10° trendelenberg tilt. For convenient operation, the surgeon would stand between the legs after the optic trocar above the pubis is established. Surgeons may gain technical and ergonomic benefits with medial trocar placement, reducing physical stress and strain while improving visualization and access to target tissues.^[11]^ Working trocars around inguinal region should be meticulously inserted to avoid damaging the inferior epigastric vessels, which can be placed on the medial or lateral of these vessels. Dissection into the retromuscular space can be very challenging in some patients, particularly in case of narrow retrorectus planes. Identification of the arcuate line contributes to enter the correct retro-rectus space. Dissection is sometimes difficult due to the maneuver angle, which would tend to enter the preperitoneal space. The key is keeping dissection close to the abdominal wall muscle under a pressing down on the body surface. During the midline dissection, the operator should gently detach the peritoneum from the linea alba and maintain its integrity; dissection of the posterior sheath should be as close to the middle line as possible to avoid tension when closing the posterior sheath. During the phase of reduction of hernial sac into the abdominal cavity, we proceed to a less extensive dissection of sac to avoid the umbilical skin injury, whereas the extraperitoneal fatty tissue should be completely reduced. Surgeons should take care not to damage both the superior and inferior epigastric vessels during dissection of bilateral retro-rectus space. Surgical needles were inserted transcutaneously to mark the edges of the fascial defects. This allowed us to measure the size of the hernias and precisely locate the mesh.^[41,42]^ The knotless barbed sutures (V-Loc; Covidien, Mansfield, MA) are recommended to close the posterior sheath. If the tension is large, the assistant can press the bilateral side abdominal wall to reduce the pressure. A firm grasp of the anatomy was required, while excessive dissection of retro-rectus space is unnecessary. In the case of small umbilical hernia, a self-fixation polypropylene mesh (Parietene ProGrip; Medtronic, Minnesota, MN) is a good choice. We prefer to roll the mesh with adhesive side anteriorly, then the mesh is introduced to the cranial of the retro-rectus space and unrolled inferiorly like the curtain, keeping the mesh adhesive to the PRS. The use of a drain is not necessary due to the fact that only one case with symptomatic seromas was observed, which do not need any further intervention.

There have been several published literature about sublay repair similar to TES. However, the nomenclature of these technologies is not uniform. Schwarz et al^[19]^ reported EMILOS (Endoscopic mini/less open sublay) technique for ventral hernia repair. A large mesh was implanted in the retromuscular space without any fixation. The indications were midline umbilical, epigastric, or incisional hernias with a coexisting rectus diastasis. Belyansky et al^[20]^ presented the application of the enhanced-view totally extraperitoneal (eTEP) technique for the repair of midline ventral and incisional hernias. It is a multicenter study and more than half cases were massive incisional hernia. For such cases the operation is very challenging even in the most experienced hands. According to our limited experience, TES should be tried from simple cases, take primary hernia for instance, to ensure the safety of the operation. Then be used to treat incisional hernias after mastering the technique.

There are several limitations of this study by its small sample size, absence of a control group, and lack of long-term follow up. It is difficult to determine that TES is the most appropriate technique for the treatment of umbilical henia. Further studies with large sample sizes are necessary to evaluate the role and significance of TES mesh repair.

## 5 Conclusion

Preliminary results of TES showed it is a safe and feasible technique for the umbilical hernias repair. Nevertheless, further analysis is mandatory to validate our findings.

## Author contributions

**Conceptualization:** Xian-fa Wang.

**Data curation:** Yu Pan.

**Investigation:** Xiao-yan Cai.

**Methodology:** Xiao-yan Cai, Ke Chen.

**Resources:** Di-yu Huang.

**Supervision:** Qi-long Chen.

**Writing – original draft:** Xiao-yan Cai, Xiao-yan Yang.

**Writing – review & editing:** Di-yu Huang, Qi-long Chen.

## Supplementary Material

Supplemental Digital Content
